# MUC1-C activates the PBAF chromatin remodeling complex in integrating redox balance with progression of human prostate cancer stem cells

**DOI:** 10.1038/s41388-021-01899-y

**Published:** 2021-06-23

**Authors:** Masayuki Hagiwara, Atsushi Fushimi, Nami Yamashita, Atrayee Bhattacharya, Hasan Rajabi, Mark D. Long, Yota Yasumizu, Mototsugu Oya, Song Liu, Donald Kufe

**Affiliations:** 1 Dana-Farber Cancer Institute, Harvard Medical School, Boston, MA USA; 2 Department of Biostatistics and Bioinformatics, Roswell Park Comprehensive Cancer Center, Buffalo, NY USA; 3 Department of Urology, Keio University School of Medicine, Tokyo, Japan

**Keywords:** Cancer stem cells, Prostate cancer, Epigenetics

## Abstract

The polybromo-associated PBAF (SWI/SNF) chromatin remodeling complex, which includes PBRM1, ARID2, and BRD7, regulates cell differentiation and genomic integrity. MUC1-C is an oncogenic protein that drives lineage plasticity in prostate cancer (PC) progression. The present work demonstrates that MUC1-C induces *PBRM1*, *ARID2*, and *BRD7* expression by the previously unrecognized E2F1-mediated activation of their respective promoters. The functional significance of the MUC1-C→PBAF pathway is supported by demonstrating involvement of MUC1-C in associating with nuclear PBAF and driving the NRF2 antioxidant gene transcriptome in PC cells. Mechanistically, MUC1-C forms a complex with NRF2 and PBRM1 on the NRF2 target *SLC7A11* gene that encodes the xCT cystine-glutamate antiporter, increases chromatin accessibility and induces SLC7A11/xCT expression. We also show that MUC1-C and PBRM1 are necessary for induction of other NRF2 target genes, including *G6PD* and *PGD* that regulate the pentose phosphate pathway. Our results further demonstrate that MUC1-C integrates activation of PBRM1 with the regulation of antioxidant genes, ROS levels, pluripotency factor expression and the cancer stem cell (CSC) state. These findings reveal a role for MUC1-C in regulating PBAF, redox balance and lineage plasticity of PC CSC progression. Our findings also uncover involvement of MUC1-C in integrating the PBAF and BAF pathways in cancer.

## Introduction

The BAF and polybromo-associated BAF (PBAF) chromatin remodeling complexes of the SWI/SNF family are essential for mammalian gene transcription and development [1]. The PBAF complex, which includes BRG1, PBRM1/BAF180, ARID2/BAF200 and BRD7, regulates cell differentiation and genomic integrity [1]. PBRM1 is a bromodomain-containing protein of importance for DNA damage-induced transcriptional repression and DNA repair [2, 3]. PBRM1 also regulates genes involved in the oxidative stress response and the induction of apoptosis [4]. In addition, PBRM1 has been associated with conferring resistance to T cell-mediated killing of tumor cells by suppressing interferon-activated gene expression [5–7]. Other studies have implicated ARID2 in regulating interferon-induced genes, providing additional support for potential involvement of PBAF in the immune response [8]. Pleotropic activities of the PBAF complex also include binding of BRD7 to BRCA1 and regulating BRCA1-mediated transcription [9]. Additionally, the BRG1 ATPase, which is common to the PBAF and BAF complexes, plays a role in targeting PBAF on chromatin and in the remodeling of nucleosomes [1, 10, 11].

The oncogenic MUC1-C protein promotes lineage plasticity in the progression of castrate resistant prostate cancer (CRPC) to neuroendocrine prostate cancer (NEPC) by driving NE dedifferentiation, self-renewal capacity, and tumorigenicity [12]. MUC1-C has been linked to hallmarks of the cancer cell by inducing the epithelial–mesenchymal transition (EMT), epigenetic reprogramming, and the cancer stem cell (CSC) state [13, 14]. MUC1-C binds directly to the MYC HLH/LZ domain and activates MYC target genes that encode components of the Polycomb Repressive Complex 1 [15, 16]. In this way, MUC1-C drives BMI1 expression and H2A ubiquitylation [15]. MUC1-C also interacts with E2F1 in inducing expression of the PRC2 components EZH2, SUZ12 and EED, and promoting H3K27 trimethylation of tumor suppressor genes [13, 17]. Involvement of MUC1-C→E2F1 signaling in epigenetic reprogramming of PC stem cells has been extended by the demonstration that this pathway activates the embryonic stem cell BAF (esBAF) complex, which includes BRG1, ARID1A, BAF60a, BAF155, and BAF170 [18]. The significance of the MUC1-C→E2F1→esBAF pathway has been supported by induction of (i) the NOTCH1 effector of CSC function, (ii) the NANOG pluripotency factor, and (iii) PC CSC self-renewal [18].

There is no known association between MUC1-C and the PBAF complex. Accordingly, studies to investigate their potential interactions were performed in MUC1-C-driven cell models of CRPC and NEPC progression [12, 18]. We report the unrecognized findings that MUC1-C→E2F1 signaling activates the PBAF components PBRM1, ARID2, and BRD7. The importance of the MUC1-C→E2F1→PBAF pathway is supported by its involvement in the regulation of NRF2 target genes, redox balance, and the PC CSC state. Our findings also support a role for MUC1-C in integrating the PBAF and BAF pathways.

## Results

### MUC1-C is necessary for PBAF expression in human cancer cells

**Fig. 1:**
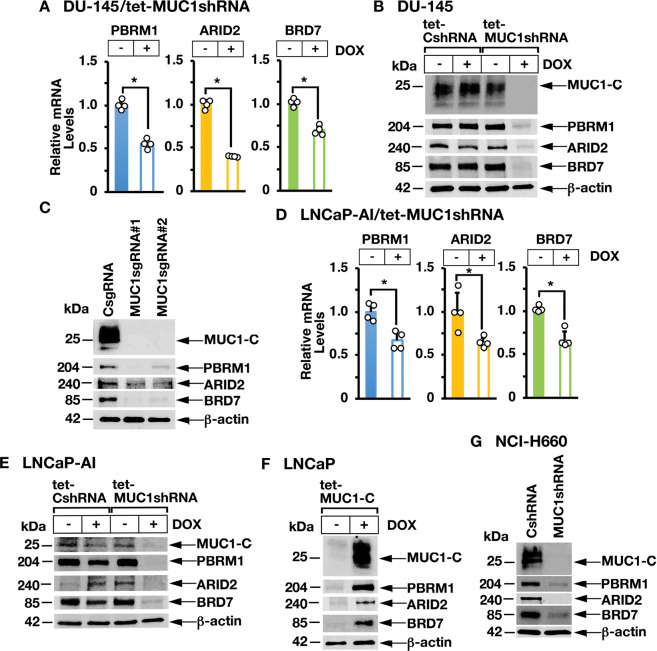
MUC1-C induces PBRM1, ARID2, and BRD7 expression. **A** DU-145/tet-MUC1shRNA cells treated with vehicle or DOX for 7 days were analyzed for PBRM1, ARID2, and BRD7 mRNA levels by qRT-PCR using primers listed in Supplementary Table S1. The results (mean ± SD of 4 determinations) are expressed as relative mRNA levels compared to that obtained for vehicle-treated cells (assigned a value of 1). **B** Lysates from DU-145/tet-CshRNA and DU-145/tet-MUC1shRNA cells treated with vehicle or DOX for 7 days were immunoblotted with antibodies against the indicated proteins. **C** Lysates from DU-145/CsgRNA, DU-145/MUC1sgRNA#1, and DU-145/MUC1sgRNA#2 cells were immunoblotted with antibodies against the indicated proteins. **D** LNCaP-AI/tet-MUC1shRNA cells treated with vehicle or DOX for 7 days were analyzed for PBRM1, ARID2, and BRD7 mRNA levels by qRT-PCR. The results (mean ± SD of 4 determinations) are expressed as relative mRNA levels compared to that obtained for vehicle-treated cells (assigned a value of 1). **E** LNCaP-AI/tet-CshRNA and LNCaP-AI/tet-MUC1shRNA cells treated with vehicle or DOX for 7 days were immunoblotted with antibodies against the indicated proteins. **F** Lysates from LNCaP/tet-MUC1-C cells treated with vehicle or DOX for 7 days were immunoblotted with antibodies against the indicated proteins. **G** Lysates from NCI-H660/CshRNA and NCI-H660/MUC1shRNA cells were immunoblotted with antibodies against the indicated proteins.

MUC1-C promotes lineage plasticity in the progression of CRPC cells [12]. The PBAF complex, which consists in part of PBRM1, ARID2, and BRD7, regulates cell differentiation [1]. To investigate whether MUC1-C regulates PBAF in association with CRPC lineage plasticity, we inducibly silenced MUC1-C in DU-145 CRPC cells and found decreases in PBRM1, ARID2, and BRD7 mRNA (Fig. 1A) and protein (Fig. 1B). Stable silencing of MUC1-C with MUC1sgRNAs (Fig. 1C) or a MUC1shRNA (Supplementary Fig. S1A) also resulted in downregulation of PBRM1, ARID2, and BRD7. Similar results were obtained in the response of androgen-independent LNCaP-AI PC cells to MUC1-C silencing (Fig. 1D, E). In support of these loss-of-function studies, induction of MUC1-C in MUC1-null LNCaP PC cells increased PBRM1, ARID2, and BRD7 expression (Fig. 1F). In addition, we found that silencing MUC1-C in (i) NCI-H660 neuroendocrine PC (NEPC) cells (Fig. 1G), (ii) BT-549 triple-negative breast cancer (Supplementary Fig. S1B) and (ii) SW620 colorectal cancer (CRC) (Supplementary Fig. S1C) cells results in downregulation of PBRM1, ARID2, and BRD7. The MUC1-C cytoplasmic domain is a 72 amino acid intrinsically disordered protein that includes a CQC motif, which is necessary for MUC1-C homodimerization and nuclear localization [13]. The cell-penetrating GO-203 peptide targets the CQC motif and blocks MUC1-C function [19]. In concert with the effects of silencing MUC1-C, treatment of LNCaP-AI, DU-145 and NCI-H660 cells with GO-203 decreased expression of PBRM1, ARID2, and BRD7 (Supplementary Fig. S1D). In addition, induction of MUC1-C with mutation of the CQC motif to AQA abrogated MUC1-C-induced PBRM1, ARID2, and BRD7 expression in MUC1-null LNCaP cells (Supplementary Fig. S1E), confirming that MUC1-C drives these PBAF components.

### MUC1-C→E2F1 signaling activates PBRM1, ARID2, and BRD7 expression

**Fig. 2:**
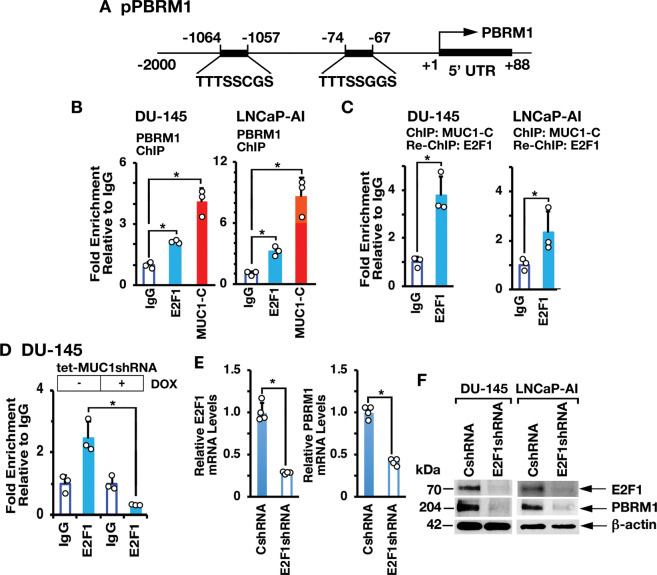
MUC1-C drives PBRM1 expression by an E2F1-mediated pathway. **A** Schema of the PBRM1 promoter region with highlighting of putative E2F binding sites. **B** Soluble chromatin from DU-145 (left) and LNCaP-AI (right) cells was precipitated with anti-E2F1, anti-MUC1-C or a control IgG. **C** Soluble chromatin from DU-145 (left) and LNCaP-AI (right) cells was precipitated with anti-MUC1-C (ChIP) and then reprecipitated with anti-E2F1 or a control IgG (re-ChIP). **D** DU-145/tet-MUC1shRNA cells were treated with vehicle or DOX for 7 days. Soluble chromatin was precipitated with anti-E2F1 or a control IgG. The DNA samples were amplified by qPCR with primers for the *PBRM1* promoter region. The results (mean ± SD of 3 determinations) are expressed as fold enrichment relative to that obtained with the IgG control (assigned a value of 1). **E** DU-145/CshRNA and DU-145/E2F1shRNA cells were analyzed for E2F1 and PBRM1 mRNA levels by qRT-PCR. The results (mean ± SD of 4 determinations) are expressed as relative mRNA levels compared to that obtained for CshRNA cells (assigned a value of 1). **F** Lysates from DU-145/CshRNA and DU-145/E2F1shRNA (left) or LNCaP-AI/CshRNA and LNCaP-AI/E2F1shRNA (right) cells were immunoblotted with antibodies against the indicated proteins.

MUC1-C directly interacts with E2F1 and contributes to activation of E2F1 target genes encoding PRC2 (EZH2, SUZ12, and EED) and BAF (BRG1, ARID1A) subunits [13, 17, 18]. There is no known association between E2Fs and the regulation of PBAF components. We identified putative E2F binding motifs in the *PBRM1* promoter (pPBRM1) (Fig. 2A) and ChIP studies of that region demonstrated occupancy by MUC1-C and E2F1 (Fig. 2B, left and right). Re-ChIP analysis further demonstrated the detection of MUC1-C/E2F1 complexes (Fig. 2C, left and right). We also found that silencing MUC1-C decreases E2F1 occupancy (Fig. 2D). In concert with these results, silencing E2F1 resulted in downregulation of PBRM1 expression (Fig. 2E, F), in support of a MUC1-C→E2F1→PBRM1 pathway.

**Fig. 3:**
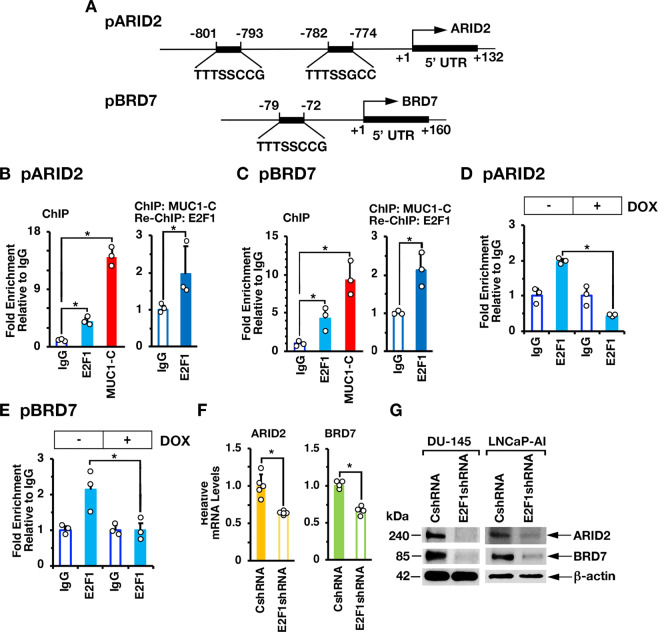
MUC1-C→E2F1 pathway induces ARID2 and BRD7 expression. **A** Schemas of the *ARID2* and *BRD7* promoter regions with positioning of putative E2F binding motifs. **B**, **C** Soluble chromatin from DU-145 cells was precipitated with anti-E2F1, anti-MUC1-C or a control IgG (left). Soluble chromatin was precipitated with anti-MUC1-C (ChIP) and then reprecipitated with anti-E2F1 or a control IgG (re-ChIP) (right). The DNA samples were amplified by qPCR with primers for the *ARID2* (**B**) and *BRD7* (**C**) promoter regions. The results (mean ± SD of 3 determinations) are expressed as the relative fold enrichment compared to that obtained with the IgG control (assigned a value of 1). **D**, **E** DU-145/tet-MUC1shRNA cells were treated with vehicle or DOX for 7 days. Soluble chromatin was precipitated with anti-E2F1 or a control IgG. The DNA samples were amplified by qPCR with primers for the *PBRM1* (**D**) and *BRD7* (**E**) promoter regions. The results (mean ± SD of 3 determinations) are expressed as the relative fold enrichment compared to that obtained with the IgG control (assigned a value of 1). **F** DU-145/CshRNA and DU-145/E2F1shRNA cells were analyzed for ARID2 and BRD7 mRNA levels by qRT-PCR. The results (mean ± SD of 4 determinations) are expressed as relative mRNA levels compared to that obtained for CshRNA cells (assigned a value of 1). **G** Lysates from DU-145/CshRNA and DU-145/E2F1shRNA (left) or LNCaP-AI/CshRNA and LNCaP-AI/E2F1shRNA (right) cells were immunoblotted with antibodies against the indicated proteins.

In extending these results, we identified putative E2F binding motifs in the *ARID2 and BRD7* promoters (Fig. 3A). ChIP studies of the *ARID2* promoter demonstrated occupancy of MUC1-C and E2F1 (Fig. 3B, left) and, as detected by re-ChIP analysis, that MUC1-C associates with E2F1 (Fig. 3B, right). Similar results were obtained in studies of the *BRD7* promoter (Fig. 3C, left and right). Silencing MUC1-C decreased E2F1 occupancy on the *ARID2* (Fig. 3D) and *BRD7* (Fig. 3E) promoters. Moreover, silencing E2F1 decreased levels of ARID2 and BRD7 mRNA (Fig. 3F) and protein (Fig. 3G). These findings collectively supported involvement of the MUC1-C→E2F1 pathway in activating PBRM1, ARID2, and BRD7 expression.

### MUC1-C and PBRM1 contribute to the regulation of NRF2 target gene signatures

**Fig. 4:**
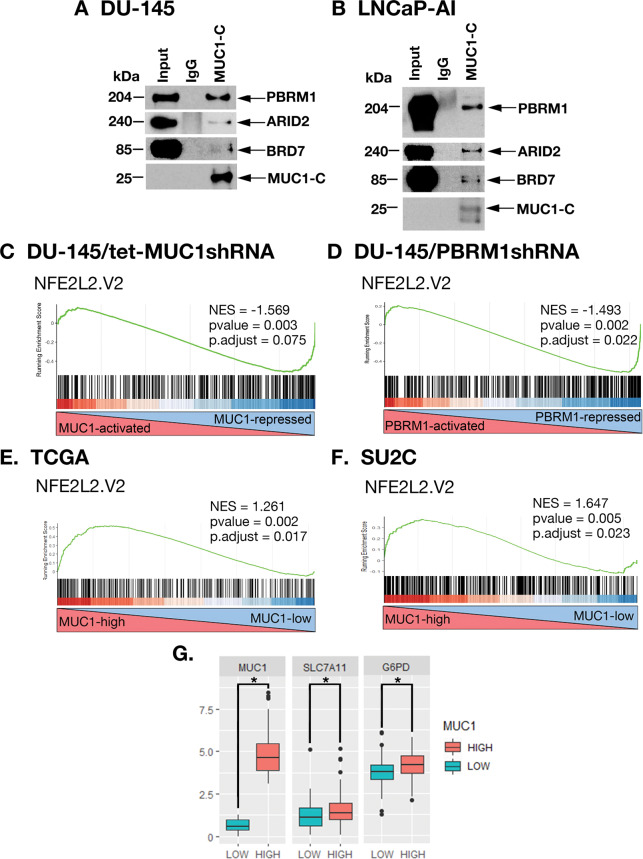
MUC1-C and PBRM1 regulate expression of NRF2 target genes in PC cells and tumors. Nuclear lysates from DU-145 (**A**) and LNCaP-AI (**B**) cells were precipitated with anti-MUC1-C or a control IgG. The precipitates and input nuclear lysates not subject to precipitation were immunoblotted with antibodies against the indicated proteins. RNA-seq was performed in triplicate on DU-145/tet-MUC1shRNA cells treated with vehicle or DOX for 7 days (**C**) and on DU-145/CshRNA and DU-145/PBRM1shRNA cells (**D**). The datasets were analyzed with GSEA using the NFE2L2.V2 gene signature. Analysis of the TCGA (**E**) and SU2C (**F**) PC datasets assessing the correlation of MUC1 with GSEA using the NFE2L2.V2 gene signature. **G** Analysis of the SU2C PC dataset assessing the correlation of MUC1 with expression of the *SLC7A11* and *G6PD* genes. The asterisk (*) denotes a *p* value < 0.05 (Wilcox-test).

In further investigating interactions between MUC1-C and PBAF, we found in nuclear co-IP studies that MUC1-C forms a complex with PBRM1, ARID2, and BRD7 (Fig. 4A, B). PBRM1 regulates reactive oxygen species (ROS) levels by activating NRF2 and antioxidant target genes [4]. MUC1-C has been linked to the regulation of the pentose phosphate pathway (PPP) [20]; however, there is no known relationship between MUC1-C and NRF2. Of interest in this regard, we found by GSEA of RNA-seq datasets that silencing MUC1-C (Fig. 4C) and PBRM1 (Fig. 4D) correlates significantly with downregulation of the NFE2L2.V2 signature derived from NRF2 target genes [21]. Consistent with involvement of the MUC1-C→E2F1→PBRM1/PBAF pathway, we found concordance of MUC1-C- and PBRM1-regulated NRF2 target genes (Supplementary Fig. S2A), which included *SLC7A11* and *G6PD*, among others that were confirmed by GSEA using the SINGH_NFE2L2_TARGETS gene signature (Supplementary Fig. S2B, C). In support of these results and importantly, analysis of the TCGA and SU2C PC tumor datasets demonstrated that MUC1 significantly correlates with activation of the NFE2L2.V2 signature (Fig. 4E, F) and *SLC7A11* and *G6PD* gene expression (Fig. 4G).

### MUC1-C→E2F1→PBRM1 signaling interacts with NRF2 to promote activation of antioxidant genes

**Fig. 5:**
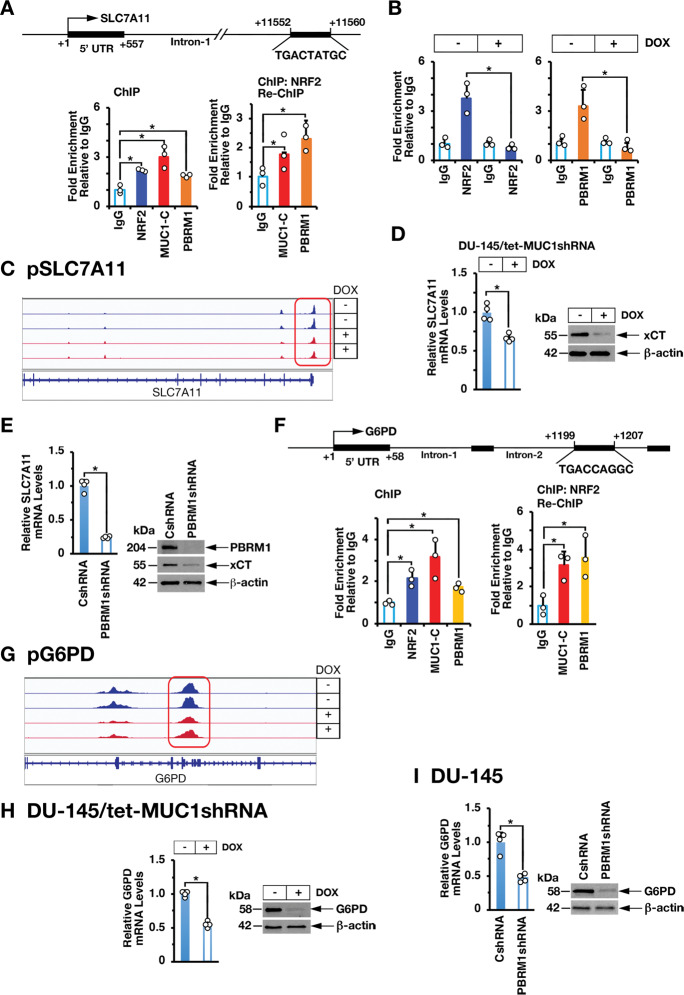
MUC1-C and PBRM1 interact with NRF2 to activate SLC7A11 and G6PD expression. **A** Schema of the *SLC7A11* promoter region with highlighting of the NRF2 binding site in intron-1. Soluble chromatin from DU-145 cells was precipitated with anti-NRF2, anti-MUC1-C, anti-PBRM1 or a control IgG (left). Soluble chromatin from DU-145 cells was precipitated with anti-NRF2 (ChIP) and then reprecipitated with anti-MUC1-C, anti-PBRM1 or a control IgG (re-ChIP). **B** Soluble chromatin from DU-145/tet-MUC1shRNA cells treated with vehicle or DOX for 7 days was precipitated with anti-NRF2 (left), anti-PBRM1 (right) or a control IgG. The DNA samples were amplified by qPCR with primers for the *SLC7A11* promoter region. The results (mean ± SD of 3 determinations) are expressed as fold enrichment relative to that obtained with the IgG control (assigned a value of 1). **C** Chromatin from DU-145/tet-MUC1shRNA cells treated with vehicle or DOX for 7 d was analyzed for ATAC-seq. UCSC genome browser snapshot of ATAC-seq data from the *SLC7A11* gene showing loss of peaks and decrease in chromatin accessibility as a function of MUC1-C silencing. **D and E.** DU-145/tet-MUC1shRNA cells treated with vehicle or DOX for 7 days (**D**) and DU-145/CshRNA and DU-145/PBRM1shRNA cells (**E**) were analyzed for SLC7A11 mRNA levels by qRT-PCR. The results (mean ± SD of 4 determinations) are expressed as relative mRNA levels compared to that obtained for CshRNA cells (assigned a value of 1)(left). Lysates were immunoblotted with antibodies against the indicated proteins (right). **F** Schema of the *G6PD* promoter region with highlighting of the NRF2 binding site in intron-2. Soluble chromatin from DU-145 cells was precipitated with anti-NRF2, anti-MUC1-C, anti-PBRM1 or a control IgG (left). Soluble chromatin from DU-145 cells was precipitated with anti-NRF2 (ChIP) and then reprecipitated with anti-MUC1-C, anti-PBRM1 or a control IgG (re-ChIP)(right). The DNA samples were amplified by qPCR with primers for the *G6PD* promoter region. The results (mean ± SD of 3 determinations) are expressed as fold enrichment relative to that obtained with the IgG control (assigned a value of 1). **G** Chromatin from DU-145/tet-MUC1shRNA cells treated with vehicle or DOX for 7 d was analyzed for ATAC-seq. UCSC genome browser snapshot of ATAC-seq data from the *G6PD* gene showing loss of peaks and decrease in chromatin accessibility as a function of MUC1-C silencing. **H and I.** DU-145/tet-MUC1shRNA cells treated with vehicle or DOX for 7 days (**H**) and DU-145/CshRNA and DU-145/PBRM1shRNA cells (**I**) were analyzed for G6PD mRNA levels by qRT-PCR. The results (mean ± SD of 4 determinations) are expressed as relative mRNA levels compared to that obtained for CshRNA cells (assigned a value of 1) (left). Lysates were immunoblotted with antibodies against the indicated proteins (right).

*SLC7A11* encodes the xCT cystine-glutamate antiporter, which as a subunit of the Xc^-^ system promotes cystine uptake for GSH synthesis and preservation of intracellular redox balance [22]. *SLC7A11* includes an NRF2 binding motif (TGACCTAGC) at positions +11552 to +11560 in intron 1 (Fig. 5A). ChIP studies of that region demonstrated occupancy of NRF2, as well as MUC1-C and PBRM1 (Fig. 5A, left). Re-ChIP experiments further demonstrated that NRF2 associates with MUC1-C and PBRM1 (Fig. 5A, right). Silencing MUC1-C decreased NRF2 (Fig. 5B, left) and PBRM1 occupancy (Fig. 5B, right) and chromatin accessibility (Fig. 5C). Moreover, silencing MUC1-C was associated with suppression of *SLC7A11* mRNA and xCT protein levels (Fig. 5D, left and right; Supplementary Fig. S3A, left and right). Similar results were obtained in response to silencing E2F1 (Supplementary Fig. S3B, left and S3C and S3D) and PBRM1 (Fig. 5E, left and right).

The PPP, which is regulated in part by glucose-6-phosphate dehydrogenase (G6PD), converts NADP+ to NADPH in maintaining redox balance [23]. *G6PD* is an NRF2 target gene with an NRF2 binding motif in intron-2 (Fig. 5F). ChIP-PCR studies demonstrated that NRF2 associates with MUC1-C and PBRM1 in occupying the *G6PD* intron-2 region (Fig. 5F, left and right). We also found that silencing MUC1-C decreases NRF2 and PBRM1 occupancy (Supplementary Fig. S3E, left and right) and chromatin accessibility (Fig. 5G). Moreover, silencing MUC1-C (Fig. 5H; Supplementary Fig. S3A), E2F1 (Supplementary Fig. S3B, right and S3C and S3D) and PBRM1 (Fig. 5I) decreased G6PD expression. Similar results were obtained for the *6-phosphogluconate dehydrogenase* (*PGD)* gene, which like *G6PD* regulates the PPP [23]; that is, (i) MUC1-C, NRF2, and PBRM1 occupy the *PGD* intron-3 region (Supplementary Fig. S4A), (ii) silencing MUC1-C decreases chromatin accessibility of the *PGD* gene (Supplementary Fig. S4B), and (iii) silencing MUC1-C (Supplementary Fig. S4C), E2F1 (Supplementary Fig. S4D) and PBRM1 (Supplementary Fig. S4E) decreases PGD expression. In further support that the MUC1-C→E2F1→PBRM1 pathway also contributes to NRF2-mediated activation of antioxidant genes, we found that silencing MUC1-C (Supplementary Fig. S5A), E2F1 (Supplementary Fig. S5B) or PBRM1 (Supplementary Fig. S5C) downregulates expression of the phase II detoxification glutathione S-transferase pi (GSTP1), heme oxygenase (HMOX1), thioredoxin (TRX), TRX2, peroxiredoxin 1 (PRDX1), PRDX2, PRDX6, glutathione peroxidase 1 (GPX1) and GPX2 (Supplementary Fig. S5D, E).

### MUC1-C→PBRM1 pathway regulates redox balance and the oxidative stress response

**Fig. 6:**
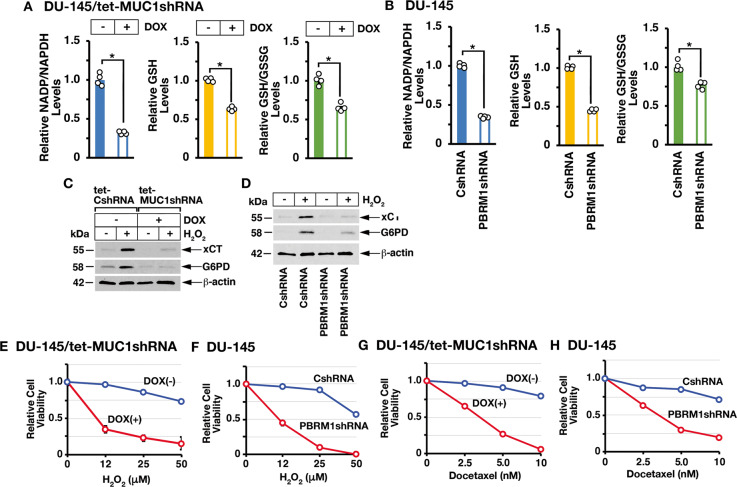
MUC1-C→PBRM1 signaling controls the oxidative stress response. **A** DU-145/tet-MUC1shRNA cells treated with vehicle or DOX for 7 days were analyzed for NADP/NADPH (left), GSH (middle), and GSH/GSSG (right) levels. The results (mean ± SD of 4 determinations) are expressed as relative levels compared to that obtained for vehicle-treated cells (assigned a value of 1). **B** DU-145/CshRNA and DU-145/PBRM1shRNA cells were analyzed for NADP/NADPH (left), GSH (middle), and GSH/GSSG (right) levels. The results (mean ± SD of 4 determinations) are expressed as relative levels compared to that obtained for CshRNA cells (assigned a value of 1). **C** DU-145/tet-CshRNA and DU-145/tet-MUC1shRNA cells were treated with DOX for 7 days and then incubated in the absence and presence of 12 μM H_2_0_2_ for 24 h. Lysates were immunoblotted with antibodies against the indicated proteins. **D** DU-145/CshRNA and DU-145/PBRM1shRNA cells were incubated in the absence and presence of 12 μM H_2_0_2_ for 24 h. Lysates were immunoblotted with antibodies against the indicated proteins. **E** DU-145/tet-MUC1shRNA cells were treated with vehicle or DOX for 7 days and then incubated in the presence of the indicated H_2_0_2_ concentrations for 24 h. **F** DU-145/CshRNA and DU-145/PBRM1shRNA cells were incubated in the presence of the indicated H_2_0_2_ concentrations for 24 h. **G** DU-145/tet-MUC1shRNA cells were treated with vehicle or DOX for 7 days and then incubated in the presence of the indicated concentrations of docetaxel for 48 h. **H** DU-145/CshRNA and DU-145/PBRM1shRNA cells were incubated in the presence of the indicated concentrations of docetaxel for 48 h. Cell viability was determined by Alamar blue staining. The results (mean ± SD of 6 determinations) are expressed as relative cell viability compared to that obtained for untreated cells (assigned a value of 1).

Consistent with involvement of MUC1-C→PBRM1 signaling in driving expression of antioxidant genes, silencing MUC1-C (Fig. 6A), E2F1 (Supplementary Fig. S6A), and PBRM1 (Fig. 6B) decreased NADP/NADPH, GSH, and GSH/GSSG levels. In extending these results, we treated cells with hydrogen peroxide (H_2_O_2_) and found that silencing MUC1-C (Fig. 6C) and PBRM1 (Fig. 6D) attenuates ROS-induced expression of xCT and G6PD (Supplementary Fig. S6B). Additionally, MUC1-C (Fig. 6E) and PBRM1 (Fig. 6F) were necessary for protection against H_2_O_2_-induced loss of cell viability. Treatment with cytotoxic agents, such as docetaxel, results in the activation of NRF2 target genes in response to disruption of DNA replication and redox balance [24]. In this context, silencing MUC1-C (Fig. 6G) or PBRM1 (Fig. 6H) increased docetaxel-induced cell death, in further support of the MUC1-C→PBRM1 pathway in regulating the oxidative stress response.

### MUC1-C→E2F1→BRG1 signaling integrates activation of the PBAF/PBRM1 and BAF/ARID1A pathways

**Fig. 7:**
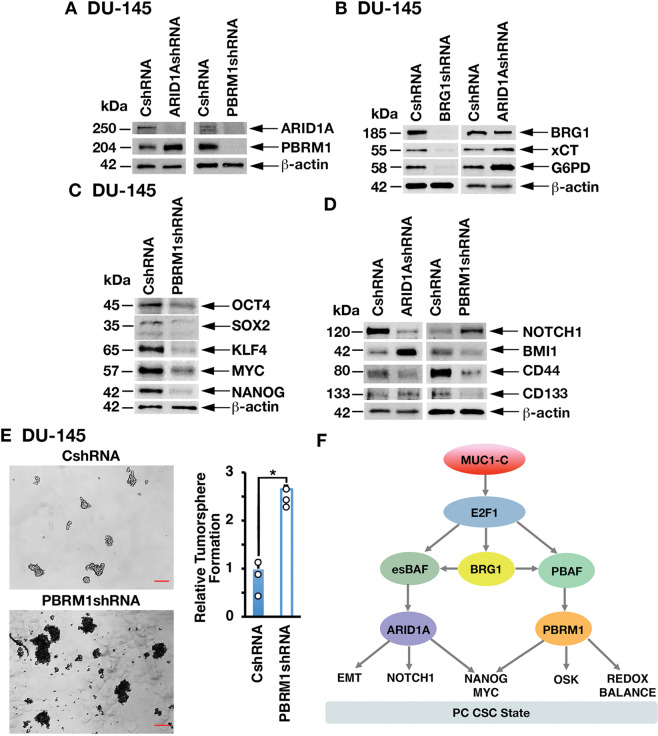
MUC1-C→BRG1 signaling integrates activation of the PBAF/PBRM1 and BAF/ARID1A pathways. **A** Lysates from DU-145/CshRNA and DU-145/ARID1AshRNA (left) or DU-145/CshRNA and DU-145/PBRM1shRNA (right) cells were immunoblotted with antibodies against the indicated proteins. **B** Lysates from DU-145/CshRNA and DU-145/BRG1shRNA (left) or DU-145/CshRNA and DU-145/ARID1AshRNA (right) cells were immunoblotted with antibodies against the indicated proteins. **C** Lysates from DU-145/CshRNA and DU-145/PBRM1shRNA cells were immunoblotted with antibodies against the indicated proteins. **D** Lysates from DU-145/CshRNA and DU-145/ARID1A (left) or DU-145/CshRNA and DU-145/PBRM1shRNA (right) cells were immunoblotted with antibodies against the indicated proteins. **E** DU-145/CshRNA and DU-145/PBRM1shRNA cells were assayed for tumorsphere formation (left). The results (mean ± SD of 3 biologic replicates) are expressed as relative tumorsphere number per field compared to the CshRNA control (assigned a value of 1) (right). Scale bar: 100 microns. **F** MUC1-C integrates activation of the esBAF and PBAF chromatin remodeling pathways in CRPC/NEPC progression. The MUC1-C cytoplasmic domain binds directly to the E2F1 DNA binding domain to activate expression of BRG1 and components of the esBAF and PBAF (PBRM1, ARID2, and BRD7) complexes. The MUC1-C→E2F1→esBAF/ARID1A pathway induces EMT and expression of NOTCH1, NANOG, and MYC. The present results demonstrate that the MUC1-C→E2F1→PBAF/PBRM1 pathway induces NRF2-mediated redox balance and expression of OSKM + NANOG. MUC1-C→E2F1→BRG1 signaling integrates cross-talk between the esBAF and PBAF pathways to drive the PC CSC state.

The BRG1 ATPase is shared by PBAF and BAF; whereas PBRM1 and ARID1A are specific components of the PBAF and BAF complexes, respectively [1]. Little is known about cross-talk between the BAF and PBAF pathways. In the present model of PC progression [12, 18], we unexpectedly found that silencing ARID1A is associated with marked upregulation of PBRM1 (Fig. 7A). As expected and like MUC1-C and PBRM1, we found that silencing BRG1 decreases xCT and G6PD expression (Fig. 7B). However, in contrast, silencing ARID1A increased xCT and G6PD levels (Fig. 7B), consistent with the associated upregulation of PBRM1. As another example of cross-talk, silencing MUC1-C suppresses the Yamanaka OSKM factors and NANOG in concert with involvement of MUC1-C in driving pluripotency [12, 18]. Silencing ARID1A was shown to induce the OSK factors [18], whereas we found here that silencing PBRM1 downregulates OSK expression (Fig. 7C). With regard to MYC and NANOG, we found that, similar to ARID1A [18], PBRM1 also drives their expression (Fig. 7C). As another example of cross-talk between the MUC1-C→BAF/ARID1A and MUC1-C→PBAF/PBRM1 pathways, silencing MUC1-C, E2F1, BRG1 and ARID1A suppressed NOTCH1 (Fig. 7D) [18] and we found that silencing PBRM1 is associated with induction of NOTCH1 expression (Fig. 7D). In addition, silencing ARID1A or PBRM1 resulted in differential regulation of the BMI1, CD44, and CD133 stemness factors (Fig. 7D). Along these lines, silencing MUC1-C, E2F1, BRG1 and ARID1A suppresses PC self-renewal capacity [18]. In contrast, the present results show that silencing PBRM1 induces tumorsphere formation (Fig. 7E), in concert with the above associated increases in ROS levels, which are linked to the regulation of CSC self-renewal capacity [25]. These findings collectively indicate that MUC1-C→E2F1→BRG1→PBAF signaling regulates redox balance, pluripotency and stemness, and integrates those functions with the MUC1-C→E2F1→BRG1→BAF pathway (Fig. 7F).

## Discussion

MUC1-C appeared in mammals to afford protection of epithelial cell layers from insults, such as infections and damage, that occur with exposure to the external environment [14]. With loss of homeostasis, MUC1-C contributes to responses of inflammation, proliferation, and remodeling that promote wound healing [14]. In this way, MUC1-C induces EMT, stemness, and epigenetic reprogramming, which if prolonged as in chronic inflammation, endow the capacity for lineage plasticity in cancer progression [12, 14, 19, 26]. The present studies were performed to investigate MUC1-C-induced pathways that promote lineage plasticity in NEPC progression [12]. Our results demonstrate that MUC1-C activates E2F1 in a pathway that induces effectors of the PBAF chromatin remodeling complex. We found that MUC1-C forms complexes with E2F1 that occupy the *PBRM1* promoter and that silencing MUC1-C decreases E2F1 occupancy and PBRM1 expression. We also found that MUC1-C and E2F1 are necessary for ARID2 and BRD7 expression, supporting a MUC1-C→E2F1→PBAF pathway in PC cells. Why MUC1-C→E2F1 signaling activates PBAF may contribute in part to the highly efficient capacity of CSCs to repair DNA damage and control ROS levels [27]. Transcriptional repression, which is necessary for DSB repair, is conferred by ATM and BMI1-mediated H2AK119 ubiquitylation [28]. MUC1-C promotes the DDR by activating ATM and integrating the BMI1→H2AK119Ub1 modification with PARP1 function [29, 30]. E2F1 is also a critical mediator of the DNA damage-induced cell cycle checkpoint response [31]. Moreover, PBRM1 is of importance for DNA damage-induced transcriptional repression and DNA repair [2, 3, 32]. Taken in the context of the present results that MUC1-C, E2F1, and PBRM1 regulate redox balance in PC cells, these findings indicate that the MUC1-C→E2F1→PBAF/PBRM1 pathway plays a role in protecting PC CSCs from ROS-induced genomic damage.

Consistent with the association between replicative and oxidative stress [33], CSCs protect against DNA damage by maintaining a robust antioxidant defense system [25]. PBAF/PBRM1 contributes to the regulation of ROS levels and induces the NRF2 target *NQO1 and HMOX1* antioxidant genes in the response to oxidative stress [4], in support of a role for PBRM1 in activating certain stress response genes to promote cell survival [4, 6]. MUC1-C also contributes to maintaining intracellular redox balance by activating the p53-inducible regulator of glycolysis and apoptosis (TIGAR), the PPP and GSH production [20, 34]. Like MUC1-C [14], NRF2 drives CSC progression and drug resistance [24]; however, there was no known association between MUC1-C and NRF2 in regulating redox balance in cancer cells. Our results demonstrate that MUC1-C regulates the NRF2 transcriptome in PC cells and, importantly, that MUC1-C and PBRM1 drive similar sets of NRF2 target genes. Among these genes, we identified *SLC7A11*, which encodes the xCT subunit of the cysteine/glutamate transporter that is regulated by MUC1-C at the cell membrane and plays an important role in GSH synthesis [35]. We found that MUC1-C associates with NRF2 and PBRM1 on the *SLC7A11* promoter and that silencing MUC1-C decreases (i) NRF2 and PBRM1 occupancy, and (ii) chromatin accessibility. In addition and like PBRM1, silencing MUC1-C suppressed constitutive SLC7A11/xCT expression. Increases in ROS induce SLC7A11/xCT expression and the present results demonstrate that MUC1-C and PBRM1 are also necessary for this oxidative stress response. In further support of the MUC1-C→PBAF/PBRM1 antioxidant pathway, MUC1-C and PBRM1 were necessary for the expression of additional redox homeostasis effectors, such as G6PD and PGD, and for induction of GSTP1, HMOX1, and other enzymes that maintain redox balance. In concert with these findings, silencing MUC1-C and PBRM1 was associated with decreases in NADPH and GSH and abrogation of the capacity to control ROS levels in the presence of oxidative and genotoxic stress.

The *BRG1* gene is frequently mutated or silenced in multiple types of cancers, indicating a role as a tumor suppressor [1, 36]. In addition, BRG1 is overexpressed as a non-mutated protein in diverse cancers and is associated with aggressive tumors in support of an oncogenic function [37, 38]. Of relevance for the present work, BRG1 has been linked to the regulation of redox balance and self-renewal capacity [39–41]. BRG1, which is common to BAF and PBAF, is induced by MUC1-C→E2F1 signaling in PC and other types of cancer cells [18]. The findings that the MUC1-C→E2F1 pathway induces BRG1, as well as the components of both esBAF and PBAF, invoked the possibility that MUC1-C→E2F1→BRG1 signaling integrates esBAF and PBAF functions (Fig. 7F). Indeed, we identified cross-talk between ARID1A and PBRM1 expression and found that MUC1-C→PBAF/PBRM1, and not MUC1-C→BAF/ARID1A, signaling induces antioxidant gene expression (Fig. 7F). Regarding lineage plasticity, MUC1-C induces the OCT4, SOX2, KLF4, and MYC pluripotency factors in NEPC progression [12]. We found that, whereas the MUC1-C→esBAF/ARID1A pathway suppresses OCT4, SOX2, and KLF4, MUC1-C→PBAF/PBRM1 drives the expression of these factors (Fig. 7F). Moreover, our findings demonstrate that the MUC1-C→esBAF and MUC1-C→PBAF pathways are both necessary for expression of MYC, as well as NANOG, which promotes stemness, treatment resistance, and poor clinical outcomes [42, 43]. In addition, the MUC1-C→esBAF/ARID1A and MUC1-C→PBAF/PBRM1 pathways differentially regulate expression of the NOTCH1, BMI1, CD44, and CD133 stemness factors and the capacity for tumorsphere formation. Taken together, these findings indicate that (i) MUC1-C→E2F1→esBAF contributes to PC CSC self-renewal, (ii) MUC1-C→E2F1→PBAF controls redox balance, which is essential for genomic stability, and (iii) both pathways regulate pluripotency factors that drive lineage plasticity (Fig. 7F). The translational relevance of these findings is underscored by MUC1-C being a druggable target that enables therapeutic options for inhibiting a network wired for maintenance of the CSC state [14].

## Materials and methods

### Cell culture

Human DU-145 (ATCC) cells were cultured in RPMI1640 medium (Corning Life Sciences, Corning, NY, USA) containing 10% heat-inactivated FBS (GEMINI Bio-Products, West Sacramento, CA, USA). Human LNCaP-AI cells were grown in phenol red-free RPMI1640 medium (ThermoFisher Scientific, Waltham, MA, USA) containing 10% charcoal-stripped FBS (Millipore Sigma, Burlington, MA, USA) [12]. Human NCI-H660 NEPC cells (ATCC) were cultured in RPMI1640 medium with 5% FBS, 10 nM β-estradiol (Millipore Sigma), 10 nM hydrocortisone, 1% insulin-transferrin-selenium (Thermo Fisher Scientific) and 2 mM L-glutamine (Thermo Fisher Scientific). Cells were treated with the MUC1-C inhibitor GO-203 [12]. Authentication of the cells was performed by short tandem repeat (STR) analysis. Cells were monitored for mycoplasma contamination using the MycoAlert Mycoplasma Detection Kit (Lonza, Rockland, ME, USA). Studies were performed on cells cultured for 3–4 months.

### Tetracycline-inducible and stable vector expression

MUC1shRNA (MISSION shRNA TRCN0000122938), E2F1shRNA (MISSION shRNA TRCN0000010328), E2F1shRNA#2 (MISSION shRNA TRCN0000039658), PBRM1shRNA (MISSION shRNA TRCN0000235890), PBRM1shRNA#2 (MISSION shRNA TRCN0000015994), BRG1shRNA (MISSION shRNA TRCN0000231102), ARID1AshRNA (MISSION shRNA TRCN0000059092), a control scrambled shRNA (CshRNA)(Millipore Sigma), and vectors encoding MUC1-C or the MUC1-C(AQA) mutant were inserted into pLKO-puro or pLKO-tet-puro (Plasmid #21915; Addgene, Cambridge, MA, USA). Guide RNA (CATCGTCAGGTTATATCGAG) targeting MUC1-C was cloned into the lentiCRISPRv2 vector (Addgene #52961). The viral vectors were produced in 293T cells [12]. Cells transduced with the vectors were selected for growth in 1–3 μg/ml puromycin. For tet-inducible vectors, cells were treated with 0.1% DMSO as the vehicle control or 500 ng/ml doxycycline (DOX; Millipore Sigma).

### Quantitative reverse-transcription PCR (qRT-PCR)

Total cellular RNA was isolated using Trizol reagent (Thermo Fisher Scientific). cDNAs were synthesized and amplified as described [12]. Primers used for qRT-PCR are listed in Supplementary Table 1.

### Immunoblot analysis

Total lysates prepared from subconfluent cells were immunoblotted with anti-MUC1-C (HM-1630-P1ABX, 1:400 dilution; ThermoFisher Scientific, Waltham, MA, USA), anti-PBRM1 (A301-591A, 1:10000; Bethyl Laboratories, Montgomery, TX, USA), anti-ARID2 (82342, 1:1000; Cell Signaling Technology (CST), Danvers, MA, USA), anti-BRD7 (15125, 1:1000; CST), anti-β-actin (A5441, 1:100,000; Sigma), anti-E2F1 (3742, 1:1000; CST), anti-SLC7A11/xCT (ab175186, 1:1000; abcam, Cambridge, MA, USA), anti-G6PD (8866, 1:1000; CST), anti-PGD (13389, 1:1000; CST), anti-BRG1 (ab110641, 1:10000; abcam, Cambridge, MA, USA), anti-ARID1A (12354, 1:500; CST), anti-OCT4 (2750, 1:1000 dilution; Cell Signaling Technology), anti-SOX2 (3579, 1:1000 dilution; Cell Signaling Technology), anti-KLF4 (12173, 1:1000 dilution; Cell Signaling Technology), anti-MYC (ab32072, 1:1000 dilution; Abcam, Cambridge, MA), anti-NANOG (4903, 1:1000; CST), anti-NOTCH1 (3608. 1:1000; CST), anti-BMI1 (6964, 1:1000, CST), anti-CD44 (KO601, 1:1000; TransGenic, Tokyo, Japan), anti-CD133 (5860, 1:1000; CST) and anti-GAPDH (5174, 1:5000, CST).

### Chromatin immunoprecipitation (ChIP) assays

Formaldehyde cross-linked and sheered soluble chromatin was precipitated with pre-cleared magnetic dynabeads (ThermoFisher Scientific) and 2 μg/ml of anti-MUC1-C **(**HM-1630-P1ABX; ThermoFisher Scientific**)**, anti-E2F1 (3742; CST), anti-NRF2 (12721; CST), anti-PBRM1 (8183; CST) or a control non-immune IgG (Santa Cruz Biotechnology). The DNA-antibody precipitates were reverse cross-linked at 65^o^C for 18 h. DNAs were purified using gel extraction columns (QIAGEN, Germantown, MD, USA) and analyzed by qPCR using the Power SYBR Green PCR Master Mix and the ABI Prism 7300 sequence detector (Applied Biosystems). Data are reported as relative fold enrichment ^12^. Primers used for ChIP qPCR are listed in Supplementary Table 2.

### ATAC-seq analysis

Assay for Transposase-Accessible Chromatin with high-throughput sequency (ATAC-seq) was performed on purified nuclei from 50,000 cells using Tn5 transposase as described [44]. Sequencing reads were used to infer regions of changes in chromatin accessibility [44].

### Tumorsphere formation assays

Cells (5 × 10^3^) were seeded per well in six-well ultra-low attachment culture plates (Corning Life Sciences) in DMEM/F12 50/50 medium (Corning Life Sciences) with 20 ng/ml EGF (Millipore Sigma), 20 ng/ml bFGF (Millipore Sigma) and 1% B27 supplement. Cells were treated with vehicle or DOX for 10–14 days. Tumorspheres were counted under an inverted microscope in triplicate wells.

Measurements of ROS, NADP/NADPH, GSH, GSH/GSSG levels. Assays for measurements of ROS (ROS-Glo H_2_O_2_ Assay, G8820; Promega, Madison, WI, USA), NADP/NADPH (NADP/NADPH-Glo Assay, G9081; Promega), GSH (GSH-Glo Glutathione Assay, V6911; Promega) and GSH/GSSG (GSH/GSSG-Glo Assay, V6611; Promega) levels were performed according to the manufacturer’s instructions validated with internal controls. Luminescence intensity was detected using an Infinite 200 PRO (Tecan, Madison, WI, USA).

### Statistical analysis

Each experiment was performed at least three times. Data are expressed as the mean ± SD. The unpaired Mann–Whitney *U* test or Student’s *t*-test was used to determine differences between means of groups. A *p* value of <0.05 denoted by an asterisk (*) was considered statistically significant.

## Supplementary information

Supplemental Material

## Data Availability

The accession number for the RNA-seq data is GEO Submission GSE139335.
