# Supplementary material for: MUC1-C activates the PBAF chromatin remodeling complex in integrating redox balance with progression of human prostate cancer stem cells
Source: Oncogene. Author manuscript; Available in PMC 2021 Aug 24. (PMC8321896; doi:10.1038/s41388-021-01899-y)
Supplement: Supplemental Material [file NIHMS1731373-supplement-Supplemental_Material.pdf]

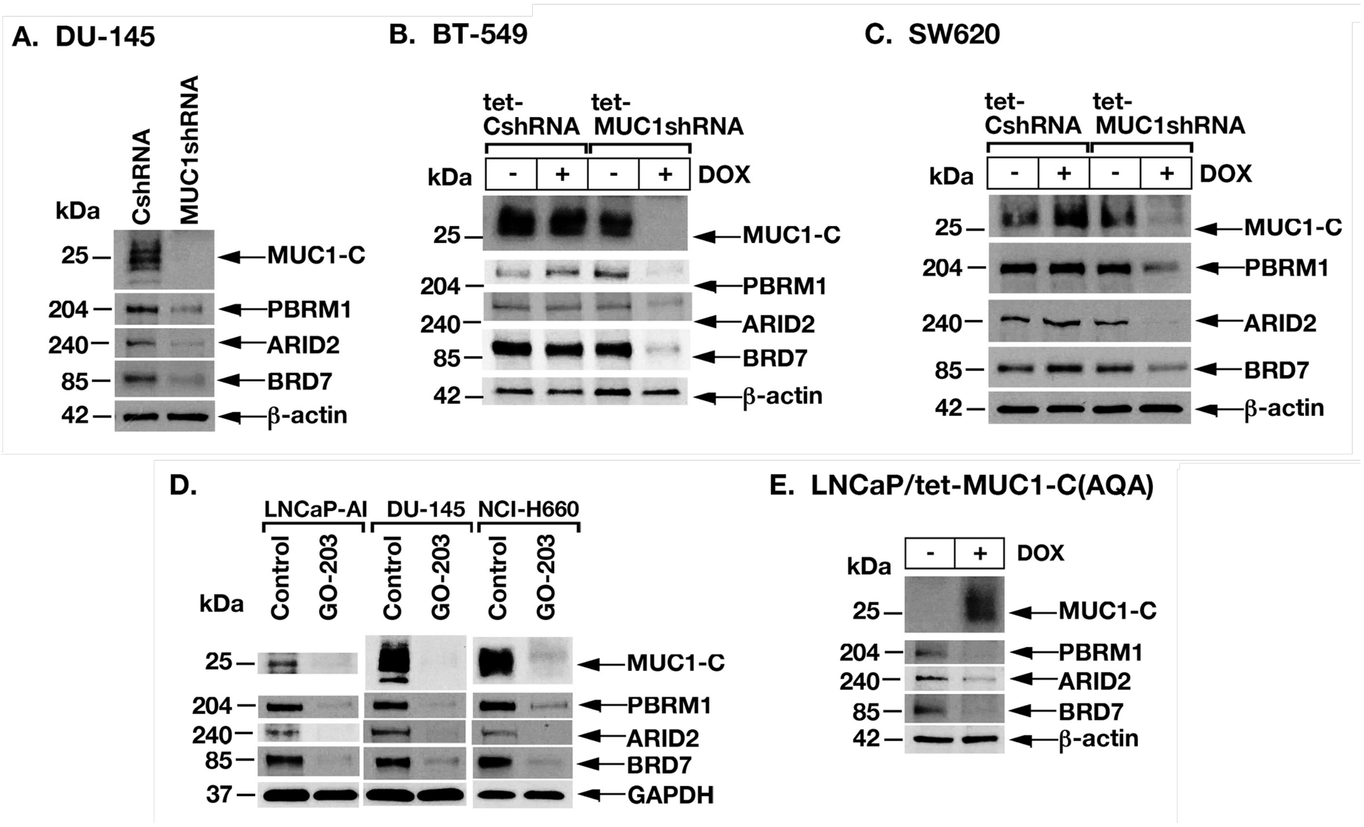

Supplemental Figure S1. Targeting MUC1-C downregulates PBRM1, ARID2 and BRD7 expression in human cancer cells. A. Lysates from DU-145/CshRNA and DU-145/MUC1shRNA cells were immunoblotted with antibodies against the indicated proteins. **B and C.** Lysates from BT-549/tet-CshRNA and BT-549/tet-MUC1shRNA (**B**) or SW620/tet-CshRNA and SW620/tet-MUC1shRNA (**C**) cells treated with vehicle or DOX for 7 days were immunoblotted with antibodies against the indicated proteins. **D.** Lysates from LNCaP-AI, DU145 and NCI-H660 cells treated with 5 μM GO-203 for 2 days were immunoblotted with antibodies against the indicated proteins. **E.** Lysates from LNCaP/tet-MUC1-C(AQA) cells treated with vehicle or DOX for 7 days were immunoblotted with antibodies against the indicated proteins.

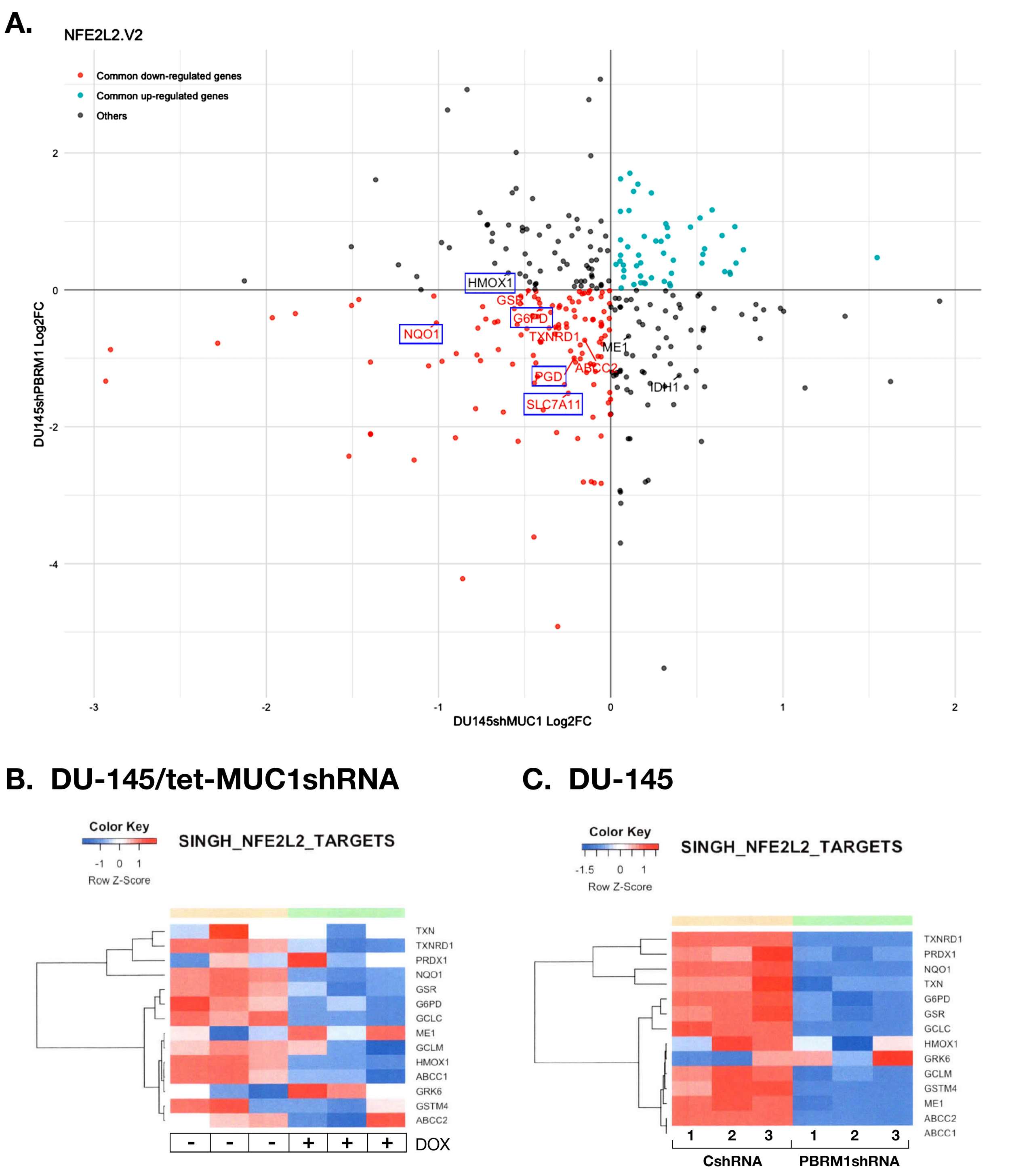

Supplemental Figure S2. MUC1-C and PBRM1 activate NRF2 gene signatures. A. Overlap of down- and up-regulated genes in DU-145 cells with MUC1-C and PBRM1 silencing obtained from GSEA of the NFE2L2.V2 gene signature. Highlighted in the blue boxes are key NRF2-induced antioxidant genes. **B and C.** Heatmaps of MUC1-C **(B)** and PBRM1 **(C)** regulated genes identified from GSEA of the SINGH NFE2L2 TARGETS gene signature.

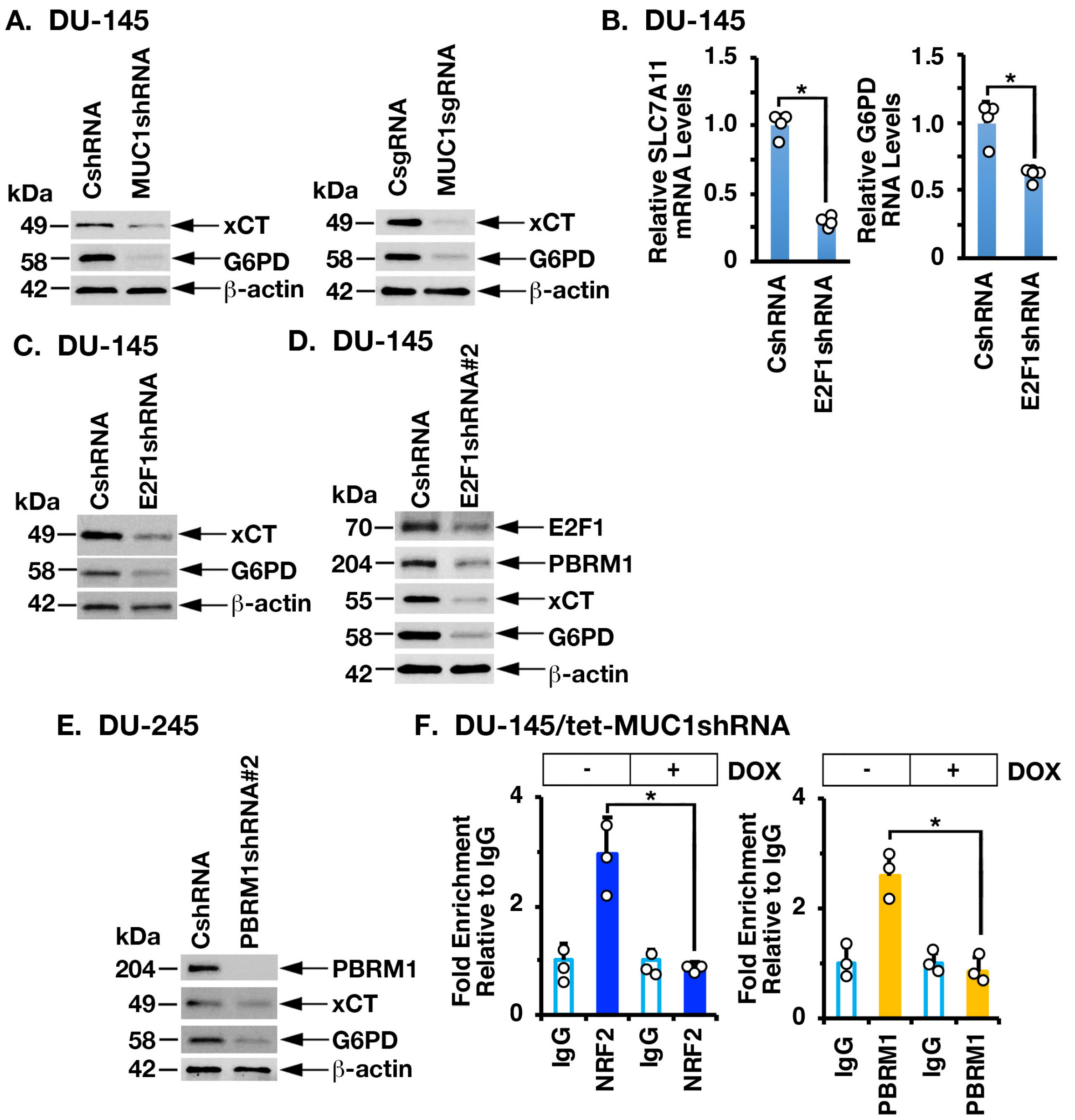

Supplemental Figure S3. Silencing E2F1 downregulates SLC7A11 and G6PD expression. A. Lysates from DU-145/CshRNA and DU-145/MUC1shRNA (left) or DU-145/CsgRNA and DU-145/MUC1sgRNA (right) cells were immunoblotted with antibodies against the indicated proteins. **B.** DU-145/CshRNA and DU-145/E2F1shRNA cells were analyzed for the indicated mRNA levels by qRT-PCR. The results (mean±SD of 3 determinations) are expressed as relative mRNA levels compared to that obtained for CshRNA cells (assigned a value of 1). **C.** Lysates were immunoblotted with antibodies against the indicated proteins. **D.** Lysates from DU-145/CshRNA and DU-145/E2F1shRNA#2 cells were immunoblotted with antibodies against the indicated proteins. **E.** Soluble chromatin from DU-145/tet-MUC1shRNA cells treated with vehicle or DOX for 7 days was precipitated with anti-NRF2 (left), anti-PBRM1 (right) or a control IgG. The DNA samples were amplified by qPCR with primers for the *G6PD*

promoter region. The results (mean±SD of 3 determinations) are expressed as fold enrichment relative to that obtained with the IgG control (assigned a value of 1).

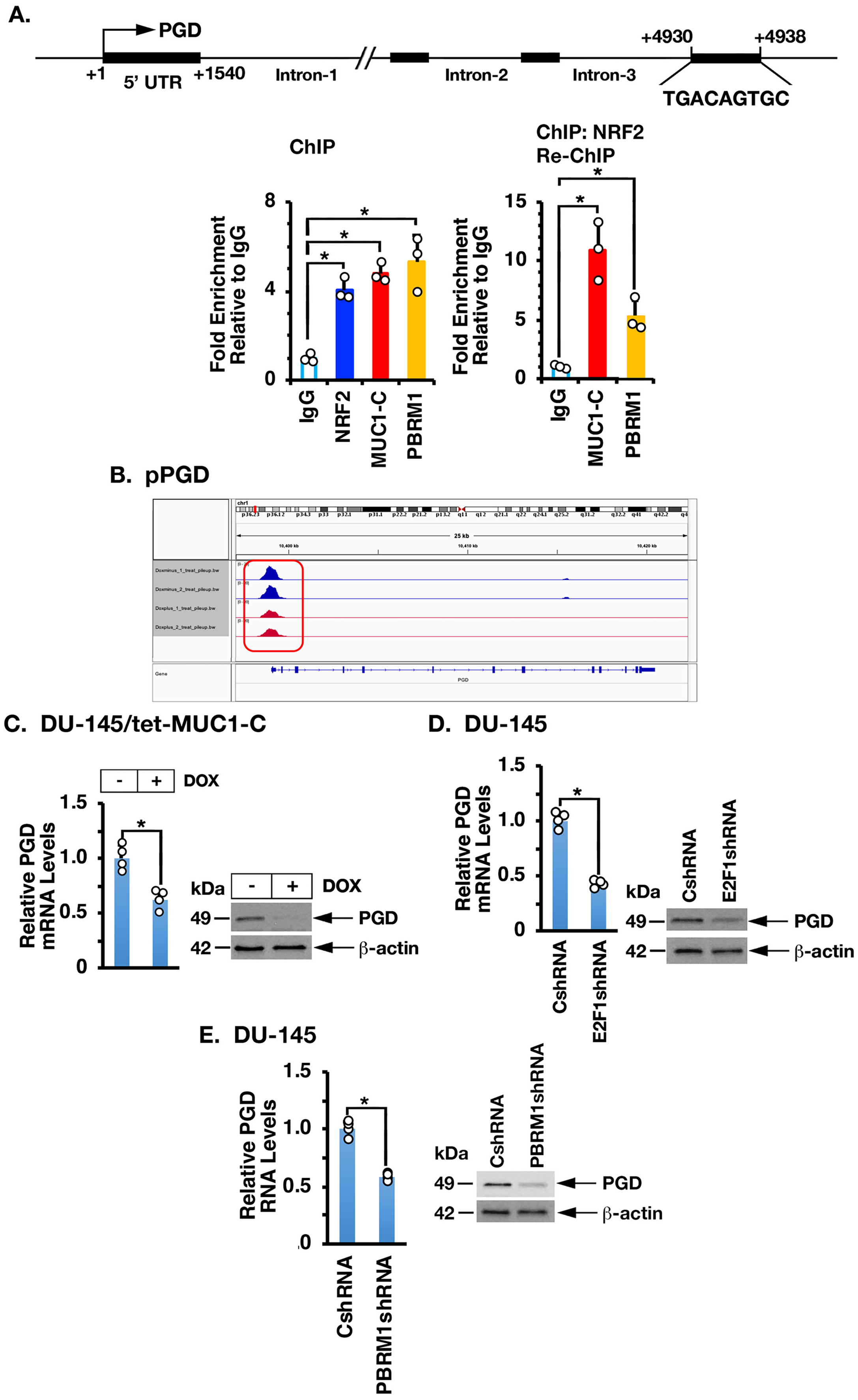
A.
PGD
+1
5' UTR
+1540
Intron-1
Intron-2
Intron-3
+4930
+4938
TGACAGTGC
ChIP
Fold Enrichment Relative to IgG
8
4
0
*
*
*
IgG
NRF2
MUC1-C
PBRM1
ChIP: NRF2
Re-ChIP
15
10
5
0
IgG
MUC1-C
PBRM1
B. pPGD
chr1
25 kb
10,400 kb
10,410 kb
10,420 kb
Gene
PGD
C. DU-145/tet-MUC1-C
- + DOX
Relative PGD mRNA Levels
1.5
1.0
0.5
0
kDa
- + DOX
49
PGD
42
β-actin
D. DU-145
Relative PGD mRNA Levels
1.5
1.0
0.5
0
CshRNA
E2F1shRNA
kDa
CshRNA
E2F1shRNA
49
PGD
42
β-actin
E. DU-145
Relative PGD RNA Levels
1.5
1.0
0.5
.0
CshRNA
PBRM1shRNA
kDa
CshRNA
PBRM1shRNA
49
PGD
42
β-actin

Supplemental Figure S4. MUC1-C and PBRM1 activate the *PGD* gene. A. Schema of the *PGD* promoter region with highlighting of the NRF2 binding site in intron-3. Soluble chromatin from DU-145 cells was precipitated with anti-NRF2, anti-MUC1-C, anti-PBRM1 or a control IgG (left). Soluble chromatin from DU-145 cells was precipitated with anti-NRF2 (ChIP) and then reprecipitated with anti-MUC1-C, anti-PBRM1 or a control IgG (re-ChIP)(right). The DNA samples were amplified by qPCR with primers for the *PGD* promoter region. The results (mean±SD of 3 determinations) are expressed as fold enrichment relative to that obtained with the IgG control (assigned a value of 1). **B.** Chromatin from DU-145/tet-MUC1shRNA cells treated with vehicle or DOX for 7 d was analyzed for ATAC-seq. UCSC genome browser snapshot of ATAC-seq data from the *PGD* gene showing loss of peaks and decrease in chromatin accessibility as a function of MUC1-C silencing. **C-E.** DU-145/tet-MUC1shRNA cells treated with vehicle or DOX for 7 days (**C**), DU-145/CshRNA and DU-145/E2F1shRNA (**D**) or DU-145/CshRNA and DU-145/PBRM1shRNA (**E**) cells were analyzed for PGD mRNA levels by qRT-PCR. The results (mean±SD of 4 determinations) are expressed as relative mRNA levels compared to that obtained for CshRNA cells (assigned a value of 1)(left). Lysates were immunoblotted with antibodies against the indicated proteins (right).

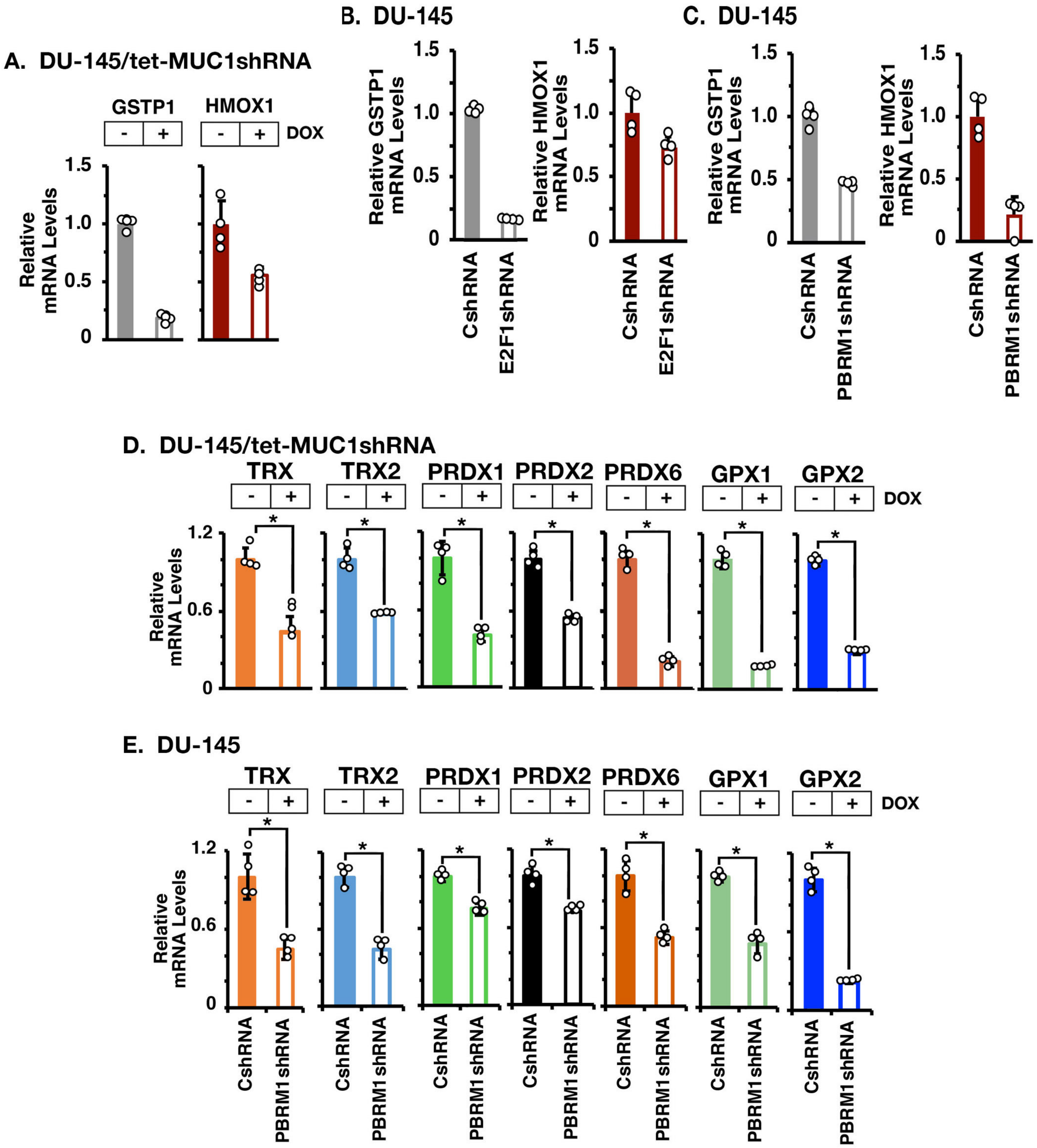

Supplemental Figure S5. MUC1-C, E2F1 and PBRM1 drive NRF2 target antioxidant genes. A. DU-145/tet-MUC1shRNA cells treated with vehicle or DOX for 7 days were analyzed for the indicated mRNA levels by qRT-PCR. The results (mean±SD of 4 determinations) are expressed as relative mRNA levels compared to that obtained for vehicle-treated cells (assigned a value of 1). **B and C.** DU-145/CshRNA, DU-145/E2F1shRNA (**B**) and DU-145/PBRM1shRNA (**C**) cells were analyzed for

the indicated mRNA levels by qRT-PCR. The results (mean±SD of 4 determinations) are expressed as relative mRNA levels compared to that obtained for CshRNA cells (assigned a value of 1). **D.** DU-145/tet-MUC1shRNA cells treated with vehicle or DOX for 7 days were analyzed for the indicated mRNA levels by qRT-PCR. The results (mean±SD of 4 determinations) are expressed as relative mRNA levels compared to that obtained for vehicle-treated cells (assigned a value of 1). **E.** DU-145/CshRNA and DU-145/PBRM1shRNA cells were analyzed for the indicated mRNA levels by qRT-PCR. The results (mean±SD of 4 determinations) are expressed as relative mRNA levels compared to that obtained for CshRNA cells (assigned a value of 1).

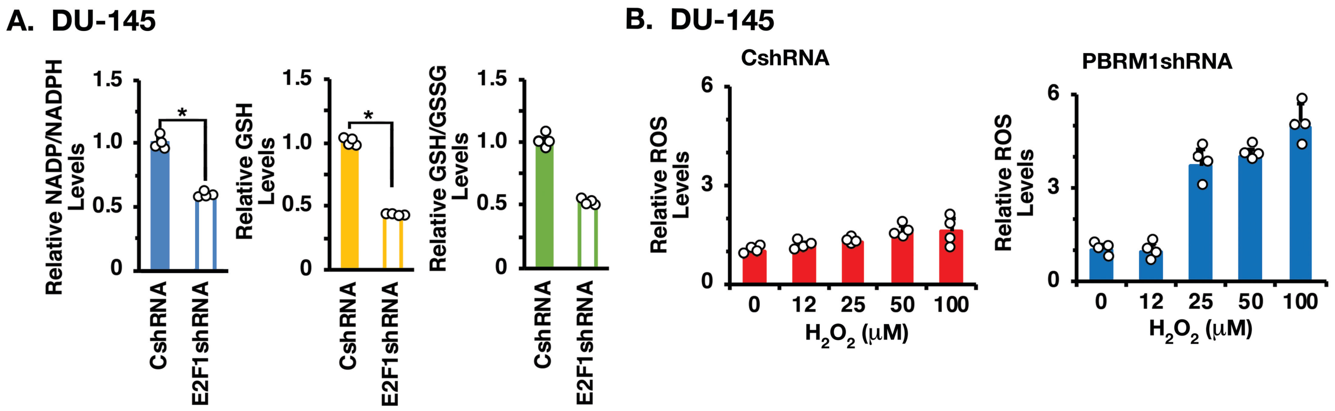

Supplemental Figure S6. Effects of silencing E2F1 and PBRM1 on redox balance. A. DU-145/CshRNA and DU-145/E2F1shRNA cells were analyzed for NADP/NADPH (left), GSH (middle) and GSH/GSSG (right) levels. The results (mean±SD of 4 determinations) are expressed as relative levels compared to that obtained for CshRNA cells (assigned a value of 1). **B.** DU-145/CshRNA (left) and DU-145/PBRM1shRNA (right) cells were incubated with the indicated H_2O_2 concentrations for 1 hour and analyzed for ROS levels. The results (mean±SD of 4 determinations) are expressed as relative ROS levels compared to that obtained for control cells (assigned a value of 1).

Supplemental Tables

Table S1. Primers used for qRT-PCR.

**MUC1-C**	**FWD**	TACCGATCGTAGCCCCTATG
	**REV**	CTCACCAGCCCAAACAGG
**PBRM1**	**FWD**	AAGAAGAAAGAGCTTGCCAG
	**REV**	TCTCGAGCTTCAAGAACAAC
**ARID2**	**FWD**	GCAGCCAATTTCCACTCCTGTTG
	**REV**	GATTGGTGACAGGAGTCCTCTG
**BRD7**	**FWD**	CAAGCTCTTTAGCCAAACAAGAA
	**REV**	TCATTCCTGAGTGCAACAGC
**E2F1**	**FWD**	TATGGTGATCAAAGCCCCTC
	**REV**	AGATGATGGTGGTGGTGACA
**SLC7A11**	**FWD**	CCATGAACGGTGGTGTGTT
	**REV**	GACCCTCTCGAGACGCAAC
**G6PD**	**FWD**	TGCCTTCCATCAGTCGGATACA
	**REV**	TGGTGGGGTAGATCTTCTTCTTGG
**PGD**	**FWD**	GGCTTTGTGGTCTGTGCTTT
	**REV**	AAATCATCCACAGCTTGCCC
**GSTP1**	**FWD**	ACCCCAGGGCTCTATGGGAA
	**REV**	TGAGGGCACAAGAAGCCCCT
**HMOX1**	**FWD**	CAGCATGCCCCAGGATTTG
	**REV**	AGCTGGATGTTGAGCAGGA
**TRX**	**FWD**	CGCGGATCCATGGTGAAGCAGATCG
	**REV**	CCGCTCGAGTTAGACTAATTCATTA
**TRX2**	**FWD**	CGCGGATCCATGGCTCAGCGACTTC
	**REV**	CCGCTCGAGTCAGCCAATCAGCTTC
**PRDX1**	**FWD**	TTTGGTATCAGACCCGAAGC
	**REV**	TCCCCATGTTTGTCAGTGAA
**PRDX2**	**FWD**	GTCCGTGCGTCTAGCCTTT
	**REV**	TCCCTTTGTAGTCCGACAGC
**PRDX6**	**FWD**	GGACGTGGCTCCCAACTTT
	**REV**	CGAGGGTGGGAGAAGAGAATG

**GPX1**	**FDW**	AAGGTACTACTTATCGAGAATGTG
	**REV**	GTCAGGCTCGATGTCAATGGTCTG
**GPX2**	**FWD**	GACACGAGGAAACCGAAGCA
	**REV**	GGCCCTTCACAACGTCT
**GAPDH**	**FWD**	CCATGGAGAAGGCTGGGG
	**REV**	CAAAGTTGTCATGGATGACC

Table S2. Primers used for ChIP-qPCR.

**pPBRM1**	**FWD**	ACTTTCTCACAGCTGCACTC
	**REV**	GCGGGAAAGTCTGGGTTAAT
**pARID2**	**FWD**	GAGCTTCCTTTCCCTTCAGAG
	**REV**	CCGGTTGTTCCAGGGTTAG
**pBRD7**	**FWD**	CAAGAAGCACAAGAAGCACAAG
	**REV**	CACTGGGAAAGAGCGGAAG
**pSLC7A11**	**FWD**	TTGACTATGCCCTGACACATTAG
	**REV**	ACAGGAAGCCATCTTCTTTCTC
**pG6PD**	**FWD**	CCTGGGTTCAAGCGATTCT
	**REV**	GGTGAAACTCCGTCTCTACTAAC
**pPGD**	**FWD**	GCGAGACTCCGTCTCAAATAA
	**REV**	ATCTACCTACAGCACACCAAC
**GAPDH**	**FWD**	TACTAGCGGTTTTACGGGCG
	**REV**	TCGAACAGGAGGAGCAGAGAGCGA
